# Action Mechanism of Rosella (*Hibiscus sabdariffa L.*) Used to Treat Metabolic Syndrome in Elderly Women

**DOI:** 10.1155/2020/5351318

**Published:** 2020-09-14

**Authors:** Yusni Yusni, Firdalena Meutia

**Affiliations:** ^1^Department of Physiology, Faculty of Medicine, Universitas Syiah Kuala, Banda Aceh 23111, Aceh, Indonesia; ^2^Department of Pharmacology, Faculty of Medicine, Universitas Syiah Kuala, Banda Aceh 23111, Aceh, Indonesia; ^3^Department of Opthalmology and Visual Science Dr. Zainoel Abidin Hospitals, Faculty of Medicine, Universitas Syiah Kuala, Banda Aceh 23111, Aceh, Indonesia

## Abstract

**Objective:**

Rosella is a safe medicinal herb used by people in Indonesia. They believe that rosella is effective in controlling metabolic syndrome, working with antihypertension, antidiabetic, antidyslipidemia and antiobesity effects. The purpose of this study was to determine the effect of rosella in controlling metabolic syndrome through the secretion of blood nitric oxide (NO) and the cortisol pathway.

**Methods:**

This study was a quasiexperimental, pretest-posttest with control group design. The total subjects were 18 people, women, and the elderly. Subjects were selected randomly into 2 groups: control group (*n* = 8) and treatment group (*n* = 8). The treatment was rosella tea, a dose of 2 grams, duration 2 times a day, given in the morning (08.00–8.30 a.m.) and evening (06.30–7.00 p.m.) after meals for 21 days. Examination of NO and cortisol levels was carried out using the enzyme-linked immunosorbent assay (ELISA) method.

**Results:**

There was a significant decrease in bodyweight (BW) (*p* = 0.021), systolic blood pressure (SBP) (*p* = 0.001), diastolic blood pressure (DBP) (*p* = 0.049), glucose preprandial (FPG) (*p* = 0.014), total cholesterol (CT) (*p* = 0.001), triglycerides (TGs) (*p* = 0.014), high-density lipoprotein (HDL) (*p* = 0.001), and low-density lipoprotein (LDL) (*p* = 0.010) after consuming rosella. NO levels were significantly increased (*p* = 0.012), whereas cortisol levels decreased significantly (*p* = 0.008) after therapy with rosella tea in elderly women.

**Conclusion:**

Rosella has shown evidence to control and lower blood pressure, blood glucose, lipid profile, and cortisol in the elderly with metabolic syndrome. Rosella is a traditional medicine that has the potential to be developed as a therapy for metabolic syndrome patients.

## 1. Introduction

The epidemic of metabolic syndrome (MS) is a major public health problem in most countries in the world [1]. The epidemic of MS is not only in developed countries but also in developing countries, including Indonesia. MS is an abnormal metabolic complex with symptoms of overweight/obesity, hypertriglyceridemia, hypercholesterolemia, low levels of HDL cholesterol, high blood pressure, insulin resistance, and hyperglycemia [2–5]. MS has an impact on increasing morbidity and mortality due to complications and comorbidities [1, 3, 5]. MS patients have twice the risk of death compared to non-MS [5, 6]. MS is a major risk factor for type 2 diabetes and cardiovascular disease [1, 3, 5]. MS is at high risk for type 2 diabetes mellitus (type 2 DM) [7].

Diabetes and hypertension are lifetime risk diseases untreated but must be controlled by taking medication regularly. Drug dependence has an impact on increasing the economic burden on families and the government, drab, and emerging fear of side effects so that patients do not want to consume drugs that tend to increase the risk of complications and comorbidities. Therefore, it is very necessary to discover traditional medicines or herbal medicine that is cheap and easily cultivable. The discovery and development of herbal medicines is a priority and crucial issue in Indonesia today. Rosella is one of the most popular herbal plants in Indonesia [8]. Rosella has long been used to treat various diseases. Rosella is a tropical plant; therefore, drab Indonesia's tropical climate is a very fertile land for growing and developing rosella.

Rosella is also one of the most popular medicinal herbs used by people in several other countries in the world [8]. Rosella is useful as an herbal medicine for antihypertension, [9, 10] antidiabetes, [11] antidyslipidemia [11], and antiobesity [12]. Flavonoids and polyphenols contained in rosella are suspected to have antidiabetic and antiobesity effects [12]. Our observations in the community show that people prefer to consume herbal medicines because they are easily obtained, of low cost, and safe for consumption. This research is a follow-up study that we conducted to analyze the other benefits of rosella besides being antihypertensive. This study aims to analyze the effect of rosella tea therapy on blood pressure, bodyweight, blood glucose levels, and plasma lipid profile. The study also analyzed the effect of rosella therapy on NO and cortisol levels in elderly women because both are thought to play a role in the pathogenesis of MS, but it is not yet clear.

NO is a potent vasodilator that functions as a vascular regulator and a central regulator of metabolism energy and body composition [13–15]. Cortisol is a hormone that has a major role in regulating metabolism and directly works in regulating blood pressure and blood glucose levels [6, 16–18]. Disorders of NO secretion and cortisol play a role in the pathogenesis of MS [6, 16–18]. We speculate that rosella plays a role in controlling MS through its work in influencing NO and cortisol secretion. We also assume that rosella possesses not only antimetabolic syndrome properties but also antistress effects. Rosella likely is developed as herbal medicine that has an impact on improving the economy of the Indonesian people.

## 2. Materials and Methods

### 2.1. Subject Research

Subjects were elderly women, aged over 60 years, with high blood pressure, high blood sugar, and dyslipidemia (high cholesterol or high triglycerides). The research sample was selected from one community, the elderly nursing home, Banda Aceh, Indonesia. Total research subjects were 18 people. The selection of research subjects was based on the results of screening with anamnesis, physical examination, and laboratory blood tests (examination of blood glucose and blood lipid profiles: total cholesterol, triglycerides, HDL, and LDL). The total population is 72 elderly men (*n* = 29) and women (*n* = 43). Based on the results of screening of 43 elderly women, it was found that as many as 18 people were experiencing metabolic syndrome (hypertension, diabetes, and dyslipidemia).

The research subjects were divided into 2 groups: the control group (*n* = 8) and the treatment group (*n* = 8). The control group was a group of elderly women treated with rosella and also antihypertensive and antidiabetic drugs. A control group is a group of elderly women who take antihypertensive and antidiabetic drugs. All subjects did not take anticholesterol drugs because all subjects were patients with diabetes and hypertension. Dyslipidemia is known by examining the lipid profile at the time of screening the patient for the study sample.

Determination of the sample for each group is done by simple random sampling using a lottery system. Hypertension criteria are determined based on JNC VII criteria. Subjects were monitored for 24 hours by nursing of home health workers. All hypertensive patients are still taking antihypertensive drugs (amlodipine), and diabetic patients take antidiabetic drugs (metformin).

### 2.2. Treatment and Laboratory Procedures

The treatment is dried rosella calyx and mixed with 150 ml of boiling water. Rosella dose is 2 grams or the equivalent of 5 calyces, given as much as 2x/day. Administration time was in the morning (8.00–8.30 a.m.) and evening (06.00–07.00 p.m.) after meals. Treatment was given for 21 days.

Examination of bodyweight (BW) was performed using a measuring scale weight (GEA ZT-120). Examination of blood pressure (BP) was performed using mercury sphygmomanometer (Riester) and stethoscope (Littmann). Measurement of blood glucose (BG) levels was used by the glucose oxidase (GOD-PAP) method. Total cholesterol (CT) was measured by the cholesterol oxidase-phenol aminophenazone (CHOD-PAP) method. Triglyceride (TG) levels were measured using an enzymatic colorimetric method with glycerol-3-phosphate oxidase-phenol aminophenazone (GPO-PAP). High-density lipoprotein (HDL) and low-density lipoprotein (LDL) levels were examined using homogeneous methods.

All examinations were carried out twice (before and after therapy): the first was done one day before giving rosella therapy (as the pretest data) and secondly done one day after rosella therapy (day 22) as the posttest data. Examination of nitric oxide and cortisol levels was carried out using the ELISA method. The patient was fasted for 12 hours from 07.00 p.m. to 07.00 a.m., before the examination. Blood samples were collected from 07.00 a.m. to 10.00 a.m.

### 2.3. Ethical Considerations

This research was approved to be carried out by the ethical standards of the research ethics committee of the Faculty of Medicine at Universitas Syiah Kuala, Banda Aceh Indonesia. This study has received written informed consent from all subjects. All information about patients is guaranteed confidentiality. The implementation of the study was by the research ethical clearance. All subject volunteers have signed informed consent before the study.

### 2.4. Statistical Analysis

Data were analyzed using parametric statistics: independent sample *t*-test and dependent sample *t*-test. Parametric statistics was carried out based on the results of an analysis of normality and homogeneity of data. The results show that the data were normally distributed and homogeneous. Statistical analysis was conducted using computer software with SPSS version 19.

## 3. Results

Subject characteristics showed that there were no differences between age, BW, BP, CT, TG, HDL, and LDL levels between control and treatment groups (*p* > 0.05), Table 1. However, there were significant differences in BG levels between the control group and the treatment group (*p* < 0.05).

Independent sample *t*-test statistics was used to determine differences in the values of the pretest and posttest data in each control and treatment group (Table 2). The results of the analysis showed that there were significant differences (*p* < 0.05) of preprandial glucose (FPG) and postprandial glucose (PPG) of pretest between the control and treatment groups. The preprandial glucose is the same as the fasting plasma glucose (FPG). There were significant differences (*p* < 0.05) in SBP, CT, and HDL posttest between the control and treatment groups. There was no difference in BW, DBP, TG, LDL, NO, and cortisol pretest and posttest between the control group and treatment group.

Analysis of paired sample *t*-tests was conducted to determine the effect of rosella tea on markers of metabolic syndrome (BW, BP, BG, CT, TG, HDL, LDL, NO, and cortisol) in Table 3. The results show that there are significant differences (*p* < 0.05) between BW (*p*=0.021), SBP, DBP, FPG, Chol, TG, HDL, LDL, NO, and cortisol before and after the administration of rosella in the treatment group. These results indicate that rosella can reduce BP in an elderly woman. Rosella reduced SBP and DBP in the elderly with hypertension. Rosella also reduces preprandial blood glucose but does not reduce PPG in the elderly with diabetes. Rosella reduced levels of CT, TG, HDL, and LDL in the elderly with dyslipidemia. Rosella lowered blood cortisol and also increased NO levels in the elderly.

## 4. Discussion

We found that rosella tea had a role in controlling MS: lowering both systolic and diastolic blood pressure in elderly women with hypertension, lowering FPG levels in elderly women with diabetes, lowering levels of Chol, TG, HDL, and LDL in elderly with dyslipidemia, and lowering BW. Rosella also affects increasing NO levels but has an inverse effect in reducing cortisol levels in the elderly with metabolic syndrome. Our results show that rosella, which has long been used by people in several countries in the world including Indonesia, is an herbal medicine that has the potential to be developed as an antimetabolic syndrome drug. Our findings were based on several scientific studies regarding the efficacy of rosella as an herbal medicine for patients with MS.

Rosella is an herb found in some countries such as Saudi Arabia, Egypt, Sudan, Mexico, Malaysia, India, Thailand, Philippines, Vietnam, and Indonesia [19]. Rosella contains phytochemicals with antihypertensive, antidyslipidemia, antiobesity, and antidiabetic effects. Rosella was safe for consumption because it has not found any side effects and adverse effects [20]. Rosella's calyx contains various chemicals such as protein (1.45%), carbohydrates (5.86%), fiber, pectin (3.19%), calcium (0.108%), phosphorus (0.052%), iron (0.021%), sodium, potassium, reducing sugars (0.82%), sucrose (0.29%), antioxidants (alkaloids, terpenoids and steroids, flavonoids, tannins and polyphenols, coumarins, saponins, glycosides, and anthraquinones), ascorbic acid, and anthocyanins [21, 22]. In Mexico and Ghana, rosella is also popular as an herbal medicine for MS: as antidiabetic, antidyslipidemia, anticholesterolemia, and antihypertensive [23–25].

Our findings regarding the potential of rosella as herbal medicines for antihypertension is supported by the theory regarding the mechanism of action of rosella in controlling blood pressure. Rosella is working in regulating cardiac output and peripheral vascular resistance, thereby reducing blood pressure and antihypertensive activity [23]. Rosella works as an angiotensin-converting enzyme (ACE) inhibitor and inhibits *α*-glucosidase and *α*-amylase, calcium channel modulation, and vasorelaxant effect [23, 26]. We found that rosella increases NO secretion. Rosella slightly increases NO levels in rats [27]. NO is a vasodilator, secreted by nitric oxide synthase (NOS) [28]. Rosella decreases lipid peroxidation and lesions in the liver, increasing the activity of the catalase and glutathione enzymes [28]. Flavonoids and anthocyanins in rosella have a diuretic effect and act as a modulator of aldosterone action and regulate blood pressure [22, 23].

Our findings regarding the efficacy of roselle tea in controlling blood lipid profiles is associated with the chemical content of rosella. Phenolic anthocyanins in rosella have antihyperlipidemia activity [27, 29]. Polyphenols have antiatherogenic, antilipogenic, and antihypertensive effects [9]. Anthocyanins, flavonoids, and polyphenols work in reducing total cholesterol and LDL [25]. Research in animal models shows that the administration of rosella seed extract for 10 weeks decreased lipid oxidation, LDL, serum cholesterol, triglycerides, and fructose. They concluded that rosella has antiatherogenic, cardioprotective, antidiabetic, and antioxidant activities [30, 31]. Rosella is reducing very low-density lipoprotein cholesterol (VLDL-c) and increasing serum HDL [26]. Rosella has a hypolipidemic effect through its pathway by inhibiting the synthesis of triacylglycerol [26].

Rosella contains some antioxidants such as flavonoids, polyphenolic acids, anthocyanins, and protocatechuic acid [26, 31]. Rosella also has cardioprotective actions [31]. Rosella decreases lipid peroxidation in the liver through the lipopolysaccharide (LPS) activity pathway [28]. LPS activates inducible nitric oxide synthase (iNOS) to stimulate NO production. iNOS is found in several places such as endothelial cells, hepatocytes cells, and Kupffer cells [28]. Polyphenols play a role in stimulating the secretion of NOS enzymes via the Pi3-K/Akt pathway [26]. Polyphenols result in relaxation of smooth muscle and activate potassium channels in a smooth muscle through nonendothelium-dependent relaxation pathways [26].

We also found that rosella tea had reduced weight in elderly women; therefore, we suspect that rosella has antiobesity effects. A study conducted on experimental animals found that roselle extract had reduced bodyweight in obese rats [26]. Rosella plays a role in the process of adipogenesis through the PI3-K/Akt and ERK pathways [26]. In vitro and in vivo studies showed that rosella increased the activity of the alpha-amylase enzyme and increased absorption and blocking sugars, thereby reducing weight [26].

We found that consumption of rosella tea decreased FPG and thus has the antidiabetic potential. Rosella acts as an antidiabetic through its action in reducing blood sugar and fasting insulin [11]; although our study did not examine plasma insulin levels, we did examine cortisol levels, which also play a role in controlling blood glucose levels. Rosella acts as an herbal medicine for type 2 diabetes and has antihyperglycemic effects through the regulation of insulin secretion [26]. Rosella is a potent inhibitor, inhibits the activity of *α*-amylase in the pancreas, and *α*-glucosidase in the intestine [19].

We found the rosella's role in lowering blood glucose levels through the cortisol pathway. Rosella lowers cortisol levels, which is a hormone that plays a role in controlling blood glucose through various mechanisms. Cortisol is a hormone that is classified into glucocorticoids that are secreted by the adrenal cortex. The function of cortisol is to control metabolism and regulate blood pressure and blood sugar [32, 33]. Cortisol works to increase gluconeogenesis and lipolysis [32]. Antioxidants play a role in inhibiting cortisol secretion [32]. Rosella is a potent antioxidant and lowers blood cortisol. Cortisol is found in several organs such as the pancreas, liver, adipose tissue, and muscle [34]. Cortisol plays a role in regulating blood glucose by regulating the activity of gluconeogenesis and glycogen synthesis [34]. This pathway is also likely to play a role in rosella activity as an antidiabetic. Decreased cortisol due to the consumption of rosella results in decreased gluconeogenesis in the liver and increased synthesis of glycogen in the liver and muscles.

## 5. Conclusion

In conclusion, rosella decreases BP (SBP and DBP), fasting blood glucose, weight, lipid profile (LDL cholesterol, total cholesterol, and triglycerides), and cortisol and increases NO levels in elderly women. We conclude that rosella has potential as an herbal drug to control metabolic syndrome. Rosella has the potential to be developed as antihypertensive, antidyslipidemic, antidiabetic, and antiobesity drugs. Rosella probably functions as an antistress herbal medicine. Further research is required to examine and analyze the mechanism of action of rosella in controlling the metabolic syndrome via a variety of cellular and molecular pathways.

## Figures and Tables

**Table 1:** Overview of subject characteristics.

Characteristics	Group	Mean ± SD	*p* value
Age (year)	Control	67.38 ± 1.99	0.16
	Treatment	67.63 ± 3.92	

Weight (kg)	Control	62.69 ± 7.74	0.42
	Treatment	64.25 ± 4.59	

SBP (mmHg)	Control	160.00 ± 7.55	0.14
	Treatment	162.50 ± 11.65	

DBP (mmHg)	Control	91.25 ± 6.40	0.34
	Treatment	88.75 ± 8.34	

Glucose (mg/dl)	Control	158.50 ± 17.76	0.001^*∗*^
	Treatment	211.88 ± 46.78	

Cholesterol (mg/dl)	Control	256.38 ± 36.59	0.43
	Treatment	242.62 ± 30.55	

TG (mg/dl)	Control	150.75 ± 165	0.86
	Treatment	165 ± 56.27	

HDL (mg/dl)	Control	50.88 ± 8.45	0.59
	Treatment	54.25 ± 7.12	

LDL (mg/dl)	Control	134.25 ± 40.16	0.63
	Treatment	139.38 ± 50.80	

^*∗*^Significantly on the level of error of 5% (*p* < 0.05).

**Table 2:** Comparison of weight, blood pressure, and blood lab before and after treatment between the control and treatment groups.

Variable	Data	Group		*p* value
		Control	Treatment	
Weight (kg)	Pretest	62.62 ± 7.74	64.25 ± 4.59	0.633
	Posttest	62.62 ± 7.84	63.38 ± 4.80	0.822

SBP (mmHg)	Pretest	160.00 ± 7.55	162.50 ± 11.65	0.620
	Posttest	160.12 ± 7.47	146.25 ± 7.90	0.003^*∗*^

DBP (mmHg)	Pretest	91.25 ± 6.40	88.75 ± 8.34	0.513
	Posttest	91.25 ± 4.43	85.62 ± 6.78	0.073

FPG (mg/dl)	Pretest	158.50 ± 17.76	211.88 ± 46.78	0.015^*∗*^
	Posttest	161.25 ± 13.62	186.88 ± 34.11	0.078

PPG (mg/dl)	Pretest	172.25 ± 59.98	278.50 ± 104.61	0.030^*∗*^
	Posttest	176.00 ± 58.60	225.25 ± 73.96	0.163

Cholesterol (mg/dl)	Pretest	256.38 ± 36.59	242.62 ± 30.55	0.429
	Posttest	265.00 ± 40.44	196.25 ± 26.76	0.002^*∗*^

TG (mg/dl)	Pretest	150.75 ± 51.80	165.00 ± 56.27	0.607
	Posttest	164.88 ± 55.68	140.62 ± 57.49	0.406

HDL (mg/dl)	Pretest	150.75 ± 51.80	165.00 ± 56.27	0.403
	Posttest	54.12 ± 8.69	36.50 ± 9.16	0.001^*∗*^

LDL (mg/dl)	Pretest	134.25 ± 40.16	139.38 ± 50.80	0.826
	Posttest	154.62 ± 41.12	115.62 ± 36.48	0.065

NO (*μ*mol/L)	Pretest	15.77 ± 2.74	16.35 ± 5.00	0.781
	Posttest	16.00 ± 2.61	18.81 ± 4.59	0.160

Cortisol (*µ*g/dl)	Pretest	16.24 ± 2.54	16.90 ± 2.95	0.352
	Posttest	15.44 ± 3.29	12.78 ± 1.56	0.066

^*∗*^Significantly on the level of error of 5% (*p* < 0.05).

**Table 3:** Effects of rosella therapy on weight, blood pressure, blood glucose levels, lipid profile, NO levels, and cortisol in the control and treatment groups.

Variable	Control group		*p* value	Treatment group		*p* value
	Pretest	Posttest		Pretest	Posttest	
Weight (kg)	62.69 ± 7.74	62.63 ± 7.84	0.59	64.25 ± 4.59	63.38 ± 4.80	0.021^*∗*^
SBP (mmHg)	160.00 ± 7.55	160.13 ± 7.47	0.92	162.50 ± 11.65	146.25 ± 7.90	0.001^*∗*^
DBP (mmHg)	91.25 ± 6.40	91.25 ± 4.43	1.00	88.75 ± 8.34	85.62 ± 6.78	0.049^*∗*^
FPG (mg/dl)	158.50 ± 17.76	161.25 ± 13.62	0.50	211.88 ± 46.78	186.88 ± 34.11	0.014^*∗*^
PPG (mg/dl)	172.25 ± 59.99	176.00 ± 58.60	0.22	278.50 ± 104.61	225.25 ± 73.96	0.077
Cholesterol (mg/dl)	256.38 ± 36.59	265.00 ± 40.44	0.32	242.62 ± 30.55	196.25 ± 26.76	0.001^*∗*^
TG (mg/dl)	150.75 ± 51.80	164.88 ± 55.68	0.26	165.00 ± 56.27	140.62 ± 57.49	0.014^*∗*^
HDL (mg/dl)	50.88 ± 8.45	54.13 ± 8.69	0.49	54.25 ± 7.12	36.50 ± 9.16	0.001^*∗*^
LDL (mg/dl)	134.25 ± 40.16	154.63 ± 41.12	0.06	139.38 ± 50.80	115 ± 36.48	0.010^*∗*^
NO (*μ*mol/L)	15.77 ± 2.74	16.00 ± 2.61	0.14	16.35 ± 5.00	18.81 ± 4.59	0.012^*∗*^
Cortisol (*µ*g/dl)	16.24 ± 0.55	15.44 ± 3.29	0.30	16.90 ± 2.95	12.78 ± 1.56	0.008^*∗*^

^*∗*^Significantly on the level of error of 5% (*p* < 0.05).

## Data Availability

The data used to support the findings of the study are available from the corresponding author on request.
